# Supplementary material for: A class of secreted mammalian peptides with potential to expand cell-cell communication
Source: Nat Commun. 2023 Dec 8;14:8125. doi: 10.1038/s41467-023-43857-0 (PMC10709327; doi:10.1038/s41467-023-43857-0)
Supplement: Supplementary file 1 — Supplementary Information [file 41467_2023_43857_MOESM1_ESM.pdf]

Supplementary Information

A class of secreted mammalian peptides with potential to expand cell-cell communication

Wiggenhorn et al.

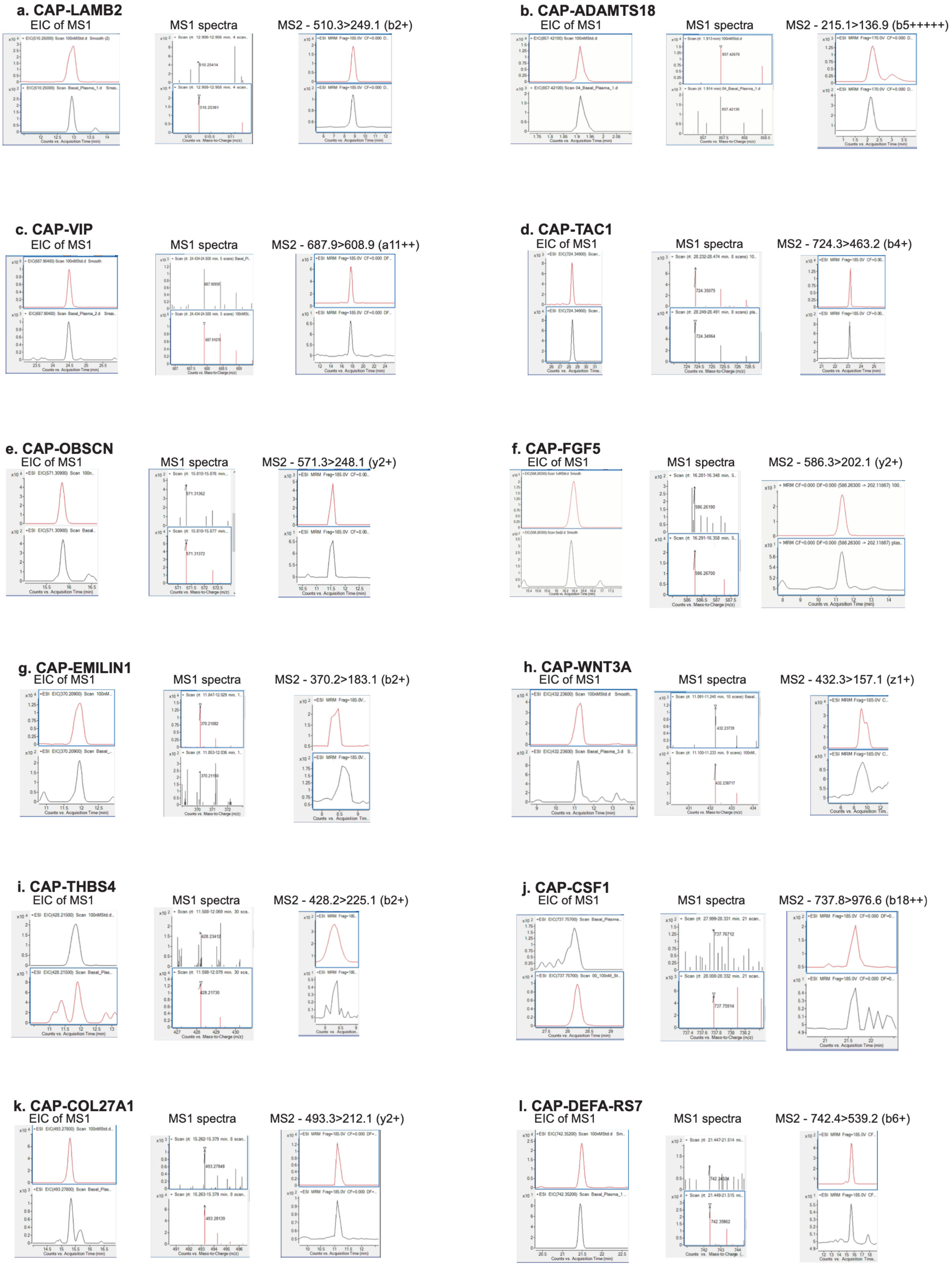

a. CAP-LAMB2
EIC of MS1
MS1 spectra
MS2 - 510.3>249.1 (b2+)
b. CAP-ADAMTS18
EIC of MS1
MS1 spectra
MS2 - 215.1>136.9 (b5+++++)
c. CAP-VIP
EIC of MS1
MS1 spectra
MS2 - 687.9>608.9 (a11++)
d. CAP-TAC1
EIC of MS1
MS1 spectra
MS2 - 724.3>463.2 (b4+)
e. CAP-OBSCN
EIC of MS1
MS1 spectra
MS2 - 571.3>248.1 (y2+)
f. CAP-FGF5
EIC of MS1
MS1 spectra
MS2 - 586.3>202.1 (y2+)
g. CAP-EMILIN1
EIC of MS1
MS1 spectra
MS2 - 370.2>183.1 (b2+)
h. CAP-WNT3A
EIC of MS1
MS1 spectra
MS2 - 432.3>157.1 (z1+)
i. CAP-THBS4
EIC of MS1
MS1 spectra
MS2 - 428.2>225.1 (b2+)
j. CAP-CSF1
EIC of MS1
MS1 spectra
MS2 - 737.8>976.6 (b18++)
k. CAP-COL27A1
EIC of MS1
MS1 spectra
MS2 - 493.3>212.1 (y2+)
l. CAP-DEFA-RS7
EIC of MS1
MS1 spectra
MS2 - 742.4>539.2 (b6+)

m. CAP-METTL24

EIC of MS1 | MS1 spectra | MS2 - 834.4>475.2 (b4+)

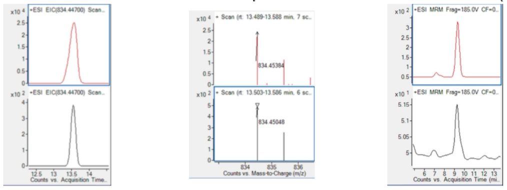

n. CAP-TENM1

EIC of MS1 | MS1 spectra | MS2 - 903.4>886.3 (z7+)

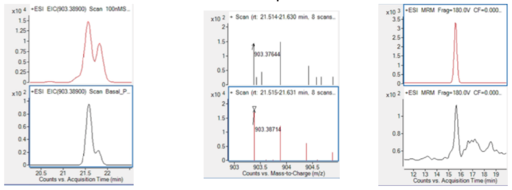

o. CAP-CA15

EIC of MS1 | MS1 spectra | MS2 - 396.2>188.1 (y2+)

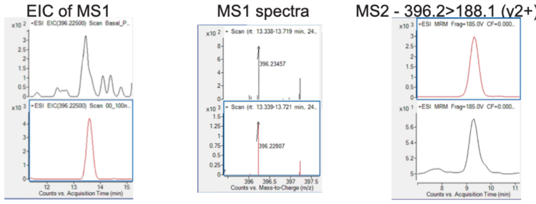

p. CAP-ANGPTL4

EIC of MS1 | MS1 spectra | MS2 - 692.4>453.2 (b4+)

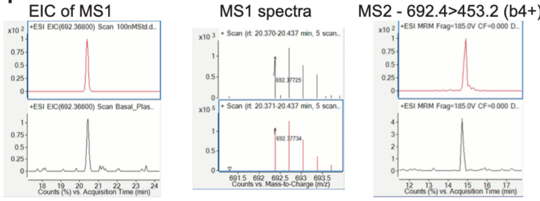

q. GnRH

EIC of MS1 | MS1 spectra | MS2 - 591.8>249.1 (b2+)

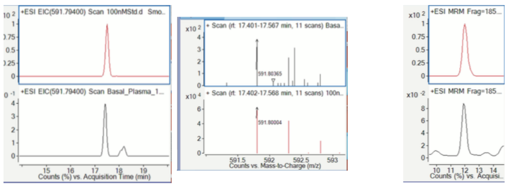

r. CAP-CNPY4

EIC of MS1 | MS1 spectra | MS2 - 557.3>439.2 (b4+)

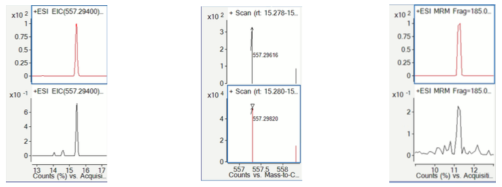

s. CAP-NTN4

EIC of MS1 | MS1 spectra | MS2 - 861.5>249.1 (b2+)

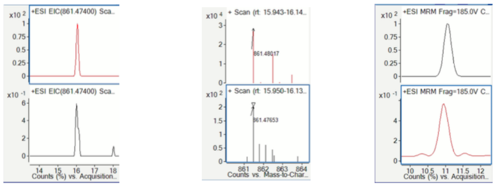

t. CAP-GDNF

EIC of MS1 | MS1 spectra | MS2 - 634.8>325.2 (b4+)

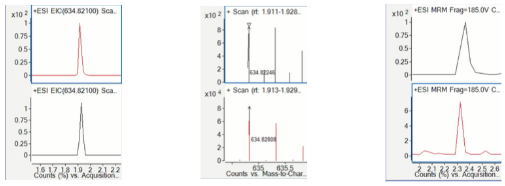

u. CAP-TAC4

EIC of MS1 | MS1 spectra | MS2 - 740.3>479.2 (b4+)

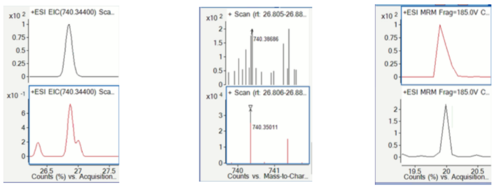

v. CAP-DEFB1

EIC of MS1 | MS1 spectra | MS2 - 1226.6>795.4 (b8+)

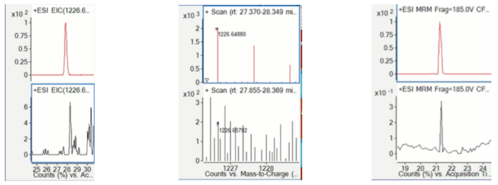

w. CAP-PRG4

EIC of MS1 | MS1 spectra | MS2 - 502.2>382.2 (y6++)

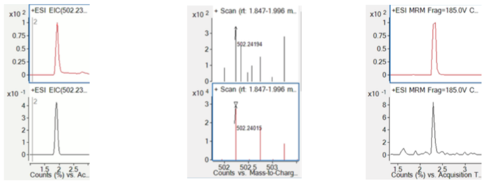

x. CAP-PLAG2G2A

EIC of MS1 | MS1 spectra | MS2 - 602.8>231.1 (a2+)

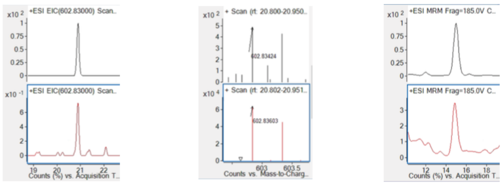

y. CAP-FGF18

EIC of MS1 | MS1 spectra | MS2 - 341.2>131.1 (y1+)

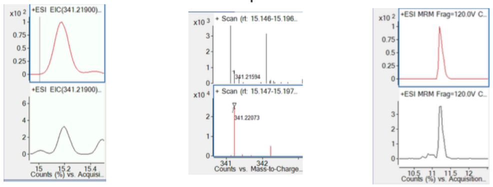

z. CAP-LY86

EIC of MS1 | MS1 spectra | MS2 - 903.4>484.3 (b4+)

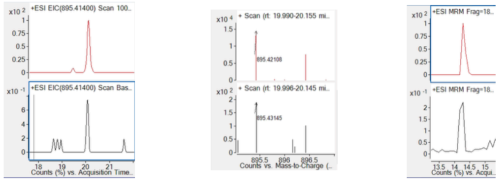

aa. CAP-VIP-2

EIC of MS1 | MS1 spectra | MS2 - 752.4>132.1 (y1+)

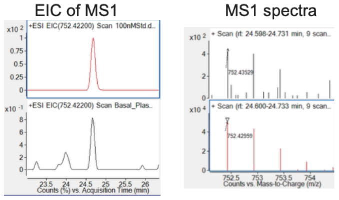

bb. CAP-PRSS36

EIC of MS1 | MS1 spectra | MS2 - 715.8>244.1 (y2+)

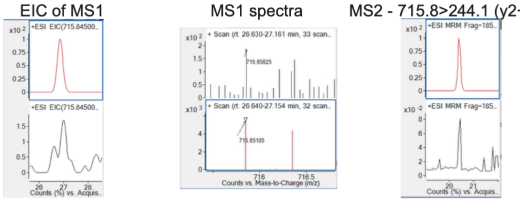

cc. CAP-GNAS

EIC of MS1 | MS1 spectra | MS2 - 693.3>325.2 (y3+)

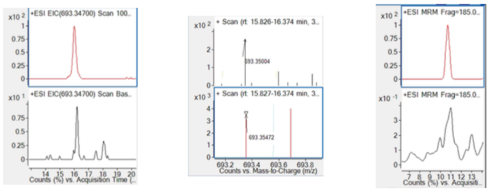

dd. CAP-GDF15

EIC of MS1 | MS1 spectra | MS2 - 682.4>354.2 (b3+)

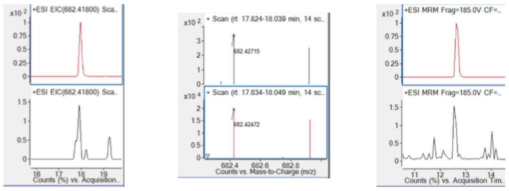

ee. CAP-TAC3

EIC of MS1 | MS1 spectra | MS2 - 803.4>729.4 (b12++)

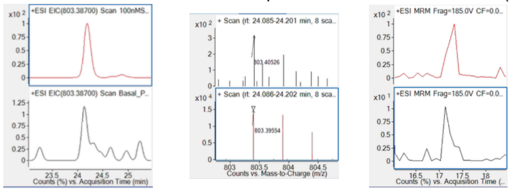

FF. CAP-EGFL8

EIC of MS1 | MS1 spectra | MS2 - 717.9>859.4 (b7+)

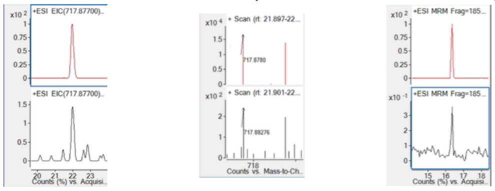

gg. CAP-COL5A2-2

EIC of MS1 | MS1 spectra | MS2 - 553.0>454.2 (b4+)

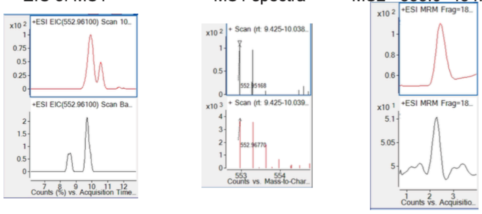

hh. CAP-MST1

EIC of MS1 | MS1 spectra | MS2 - 891.4 >306.2 (y2+)

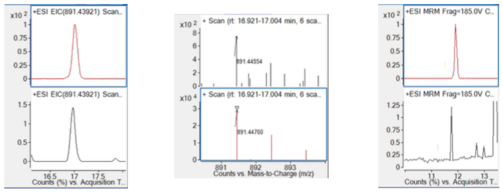

ii. CAP-ALKAL2

EIC of MS1 | MS1 spectra | MS2 - 1074.5>944.4 (b9+)

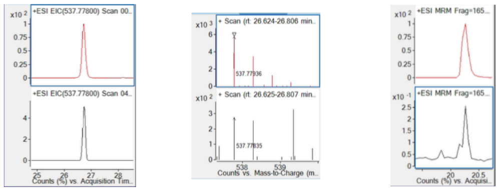

jj. CAP-GDF6

EIC of MS1 | MS1 spectra | MS2 - 661.4>209.1 (b2+)

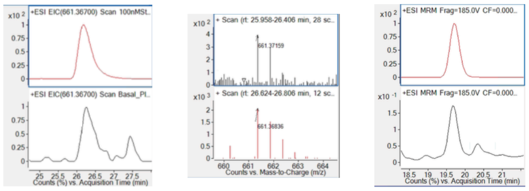

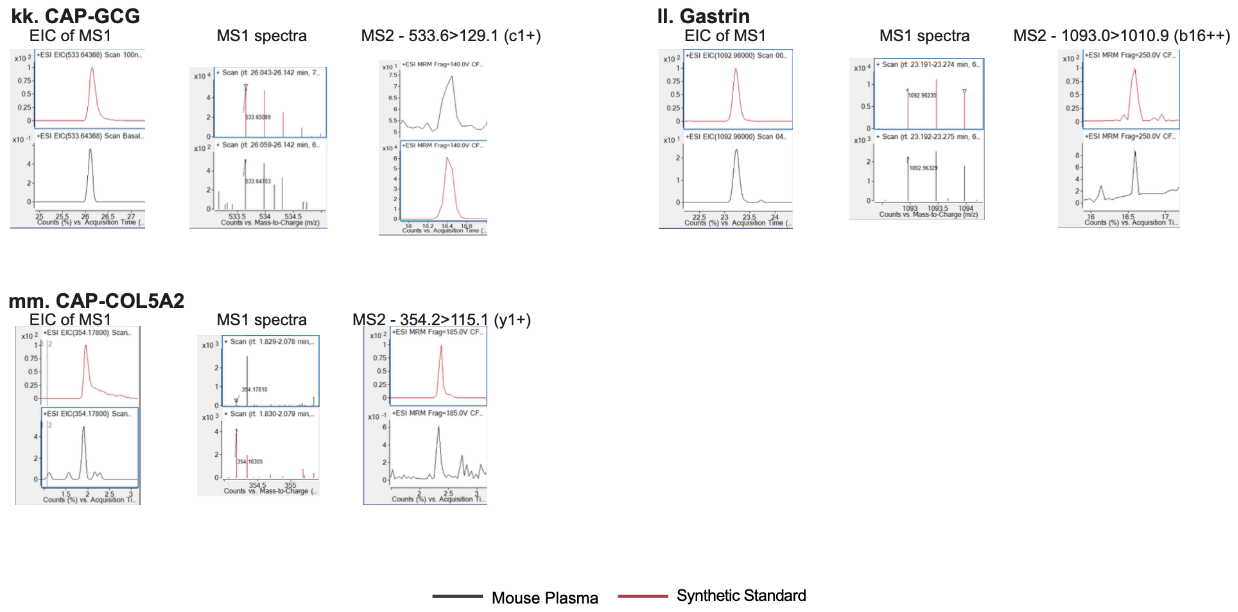

Supplementary Figure 1. All raw MS1 chromatograms, MS1 spectra, and MRM transition chromatograms for detected mouse capped peptides. a-mm The extracted parent ion chromatogram, the MS1 spectra, and MRM fragment ion chromatogram both in mouse plasma (black) and of the synthetic standards (red).

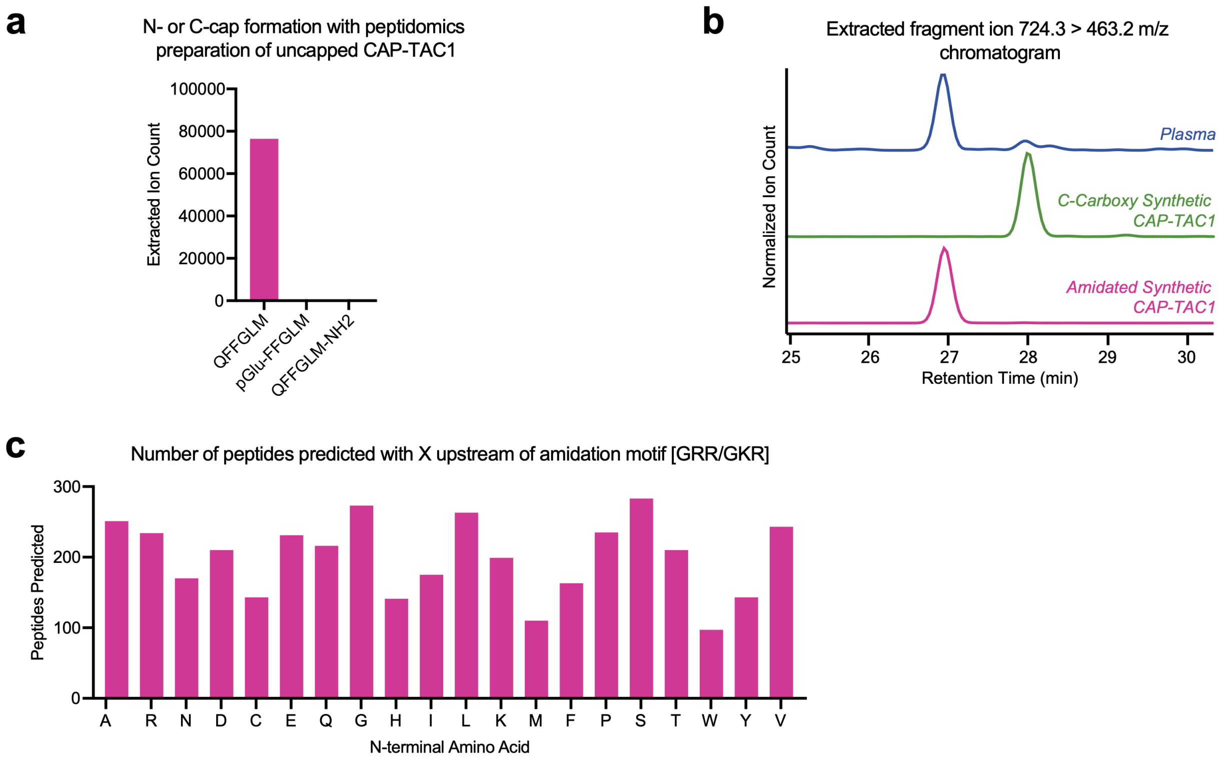

Supplementary Figure 2. Additional capped peptide prediction analysis and validation of capped peptide extraction and detection. **a** Extracted ion count of QFFGLM (uncapped CAP-TAC1, m/z = 742.359), pGlu-FFGLM (m/z = 725.33), or QFFGLM-NH2 (m/z = 741.375) after peptidomics preparation of 10 nM QFFGLM (uncapped) synthetic standard. **b** Chromatograms of CAP-TAC1 b4+ ion (724.3>463.2) in mouse plasma, 100 nM C-carboxy synthetic CAP-TAC1 standard, and 100 nM C-amidated synthetic CAP-TAC1. **c** Number of peptides predicted when the capped peptide prediction algorithm modified for X N-terminal amino acid upstream of the amidation motif. Source data are provided as a Source D ata file.

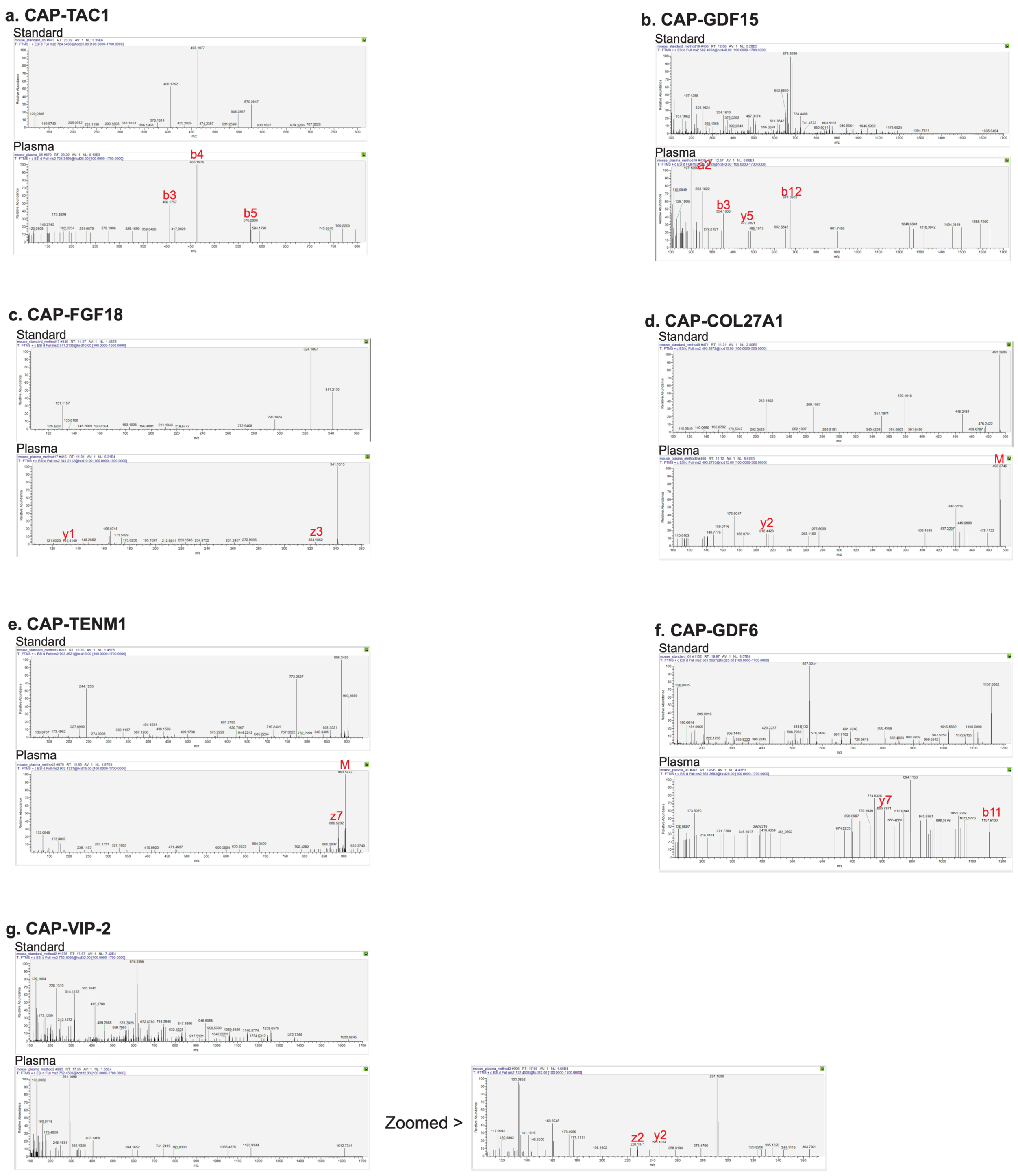

a. CAP-TAC1
Standard
Plasma
b4
b3
b5
b. CAP-GDF15
Standard
Plasma
a2
b12
b3
y5
c. CAP-FGF18
Standard
Plasma
y1
z3
d. CAP-COL27A1
Standard
Plasma
M
y2
e. CAP-TENM1
Standard
Plasma
M
z7
f. CAP-GDF6
Standard
Plasma
y7
b11
g. CAP-VIP-2
Standard
Plasma
Zoomed >
z2
y2

h. CAP-ALKAL2

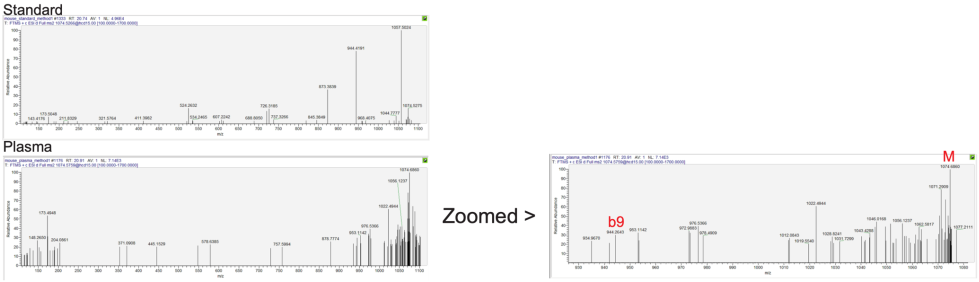

i. CAP-MST1

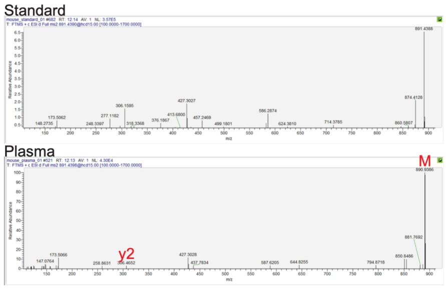

Supplementary Figure 3. Additional MS2 spectra for mouse capped peptides. a-i MS2 spectra of 9 mouse capped peptides both in mouse plasma and of the synthetic standards.

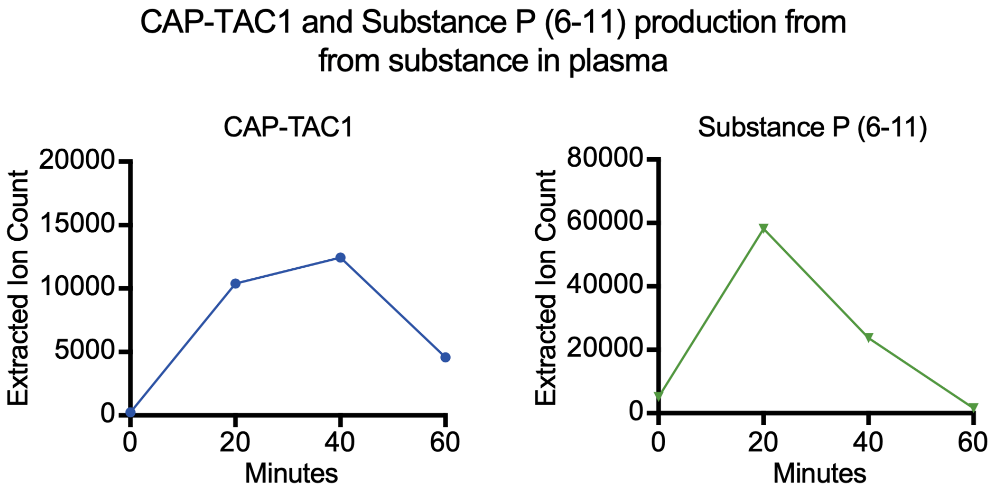

Supplementary Figure 4. CAP-TAC1 and substance P (6-11) production during substance P degradation. Relative levels of CAP-TAC1 and substance P (6-11) in mouse plasma following incubation at 37ºC for the indicated time. N=2 biological replicates per concentration per time point. Data are shown as means. Source data are provided as a Source Data file.

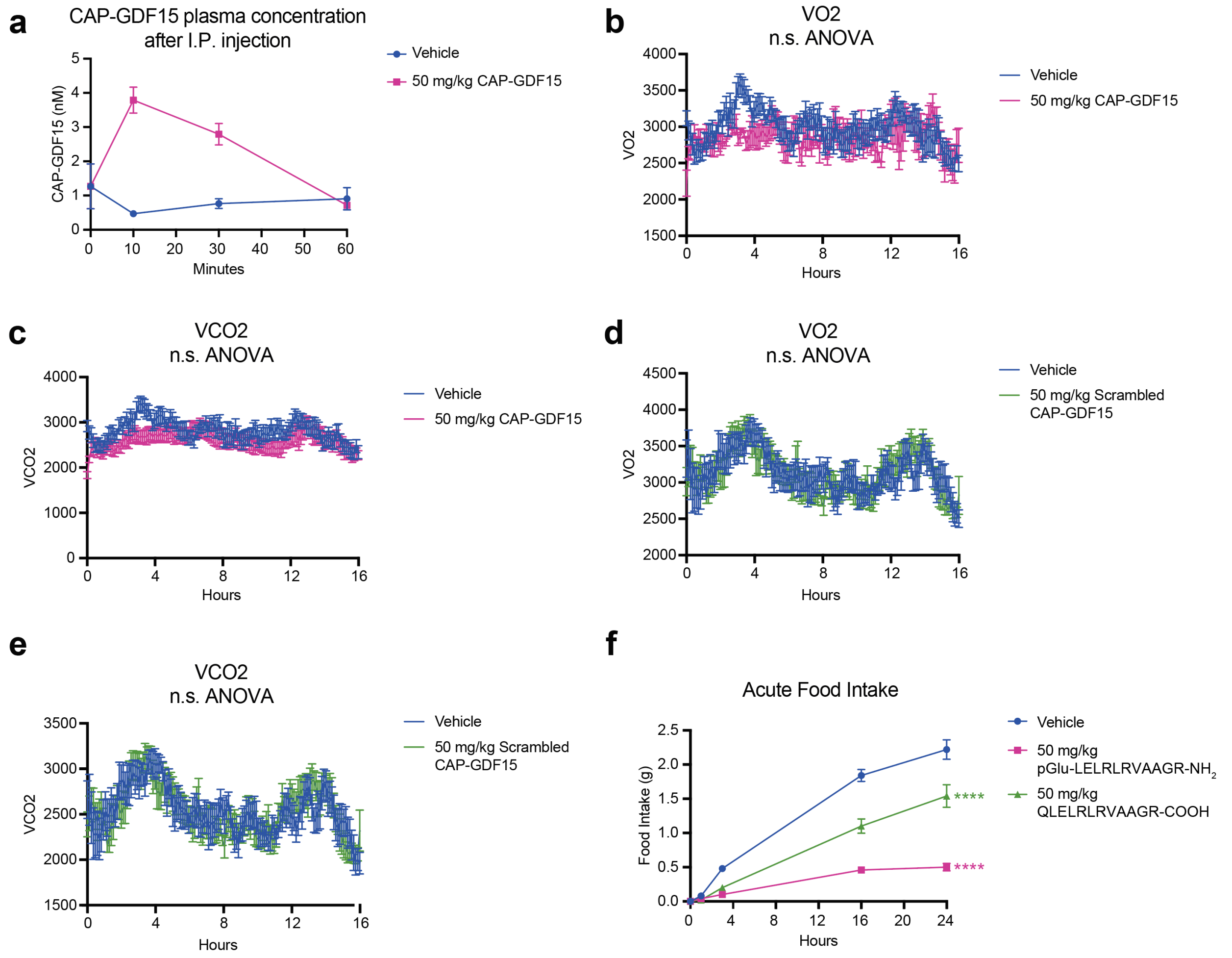

Supplementary Figure 5. Additional measurements from metabolic chambers of mice treated with CAP-GDF15. **a** CAP-GDF15 plasma concentration after administration of CAP-GDF15 (50 mg/kg, intraperitoneal) or vehicle control. **b-c** VO2 (b) and VCO2 (c) of 12-16-week diet-induced obese male mice following a single treatment of CAP-GDF15 (50 mg/kg, intraperitoneal) or vehicle control. **d-e** VO2 (**d**) and VCO2 (**e**) of 12-16-week diet-induced obese male mice following a single treatment of scrambled CAP-GDF15 (50 mg/kg, intraperitoneal, scrambled sequence: pQGLEALRARLRV-NH2) or vehicle control. **f** Acute food intake of 12-16-week diet-induced obese male mice following a single treatment of CAP-GDF15 (50 mg/kg, intraperitoneal), uncapped CAP-GDF15 (50 mg/kg, intraperitoneal), or vehicle control. For (**a**), N=3/group; (**b-c**), N=12/group; for (d-e), N=8/group; for (f), N=5/group. For (a-f), injection occurred at time T=0 (5:00pm). Data are shown as means ± SEM. For (b-i), p values were calculated by two-way ANOVA. Source data are provided as a Source Data file.

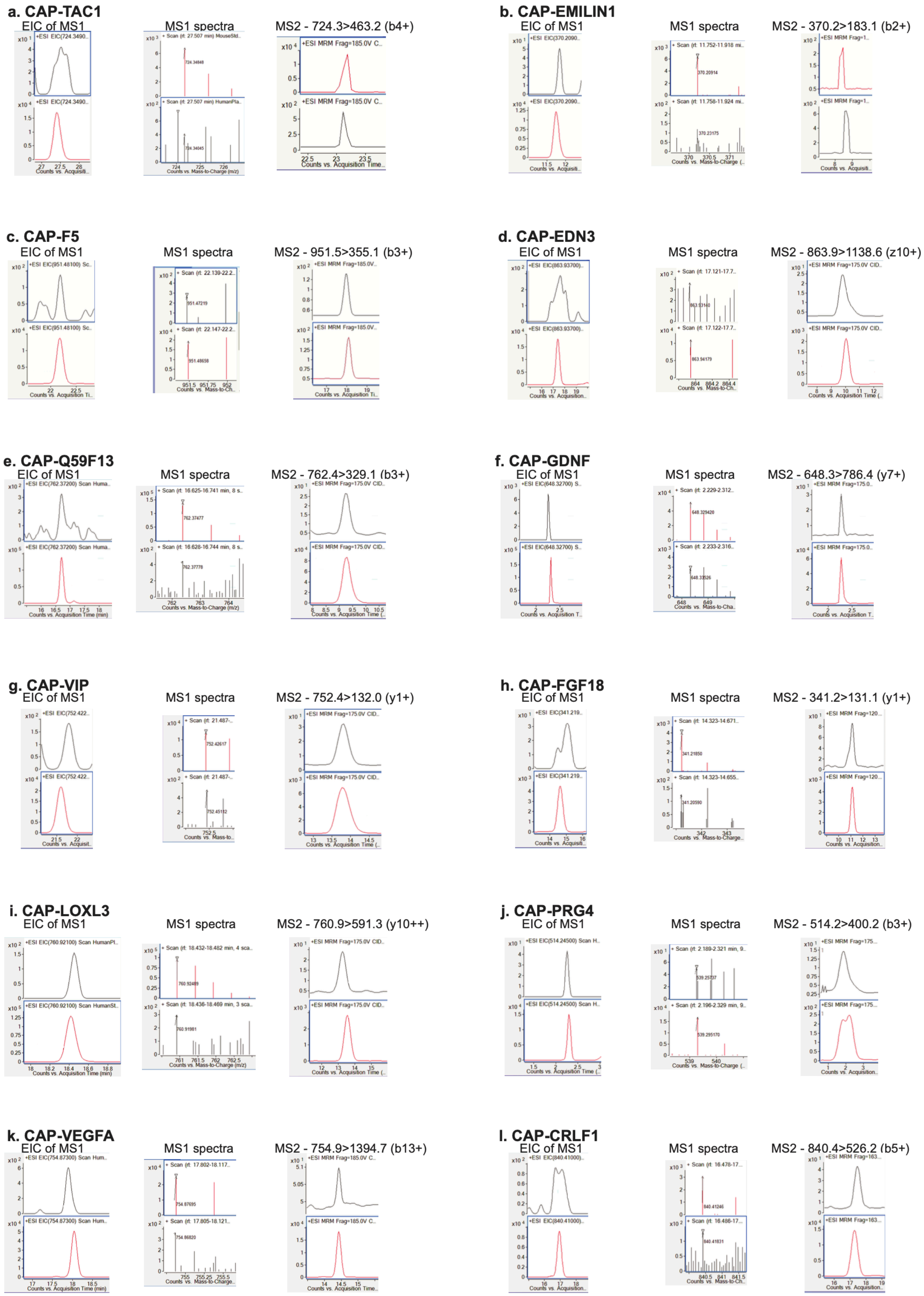

a. CAP-TAC1
EIC of MS1
MS1 spectra
MS2 - 724.3>463.2 (b4+)
b. CAP-EMILIN1
EIC of MS1
MS1 spectra
MS2 - 370.2>183.1 (b2+)
c. CAP-F5
EIC of MS1
MS1 spectra
MS2 - 951.5>355.1 (b3+)
d. CAP-EDN3
EIC of MS1
MS1 spectra
MS2 - 863.9>1138.6 (z10+)
e. CAP-Q59F13
EIC of MS1
MS1 spectra
MS2 - 762.4>329.1 (b3+)
f. CAP-GDNF
EIC of MS1
MS1 spectra
MS2 - 648.3>786.4 (y7+)
g. CAP-VIP
EIC of MS1
MS1 spectra
MS2 - 752.4>132.0 (y1+)
h. CAP-FGF18
EIC of MS1
MS1 spectra
MS2 - 341.2>131.1 (y1+)
i. CAP-LOXL3
EIC of MS1
MS1 spectra
MS2 - 760.9>591.3 (y10++)
j. CAP-PRG4
EIC of MS1
MS1 spectra
MS2 - 514.2>400.2 (b3+)
k. CAP-VEGFA
EIC of MS1
MS1 spectra
MS2 - 754.9>1394.7 (b13+)
l. CAP-CRLF1
EIC of MS1
MS1 spectra
MS2 - 840.4>526.2 (b5+)

m. CAP-FURIN

EIC of MS1 | MS1 spectra | MS2 - 912.5>1363.7 (b11+)

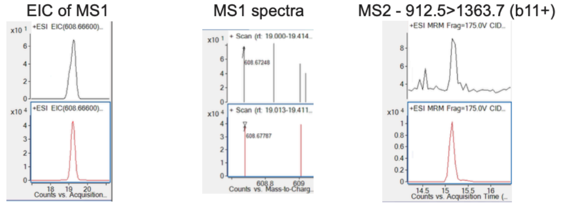

n. CAP-TG

EIC of MS1 | MS1 spectra | MS2 - 937.5>678.3 (b7+)

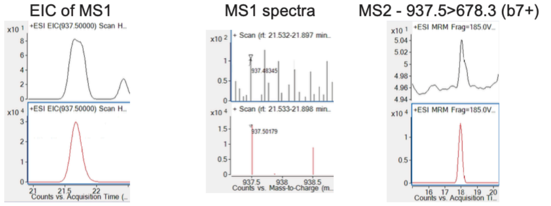

o. CAP-MEGF6-2

EIC of MS1 | MS1 spectra | MS2 - 684.4>455.2 (b4+)

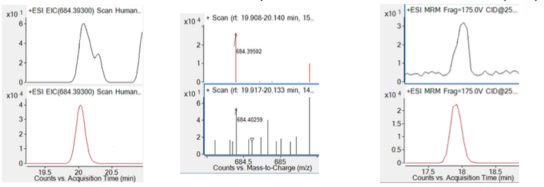

p. CAP-IFNG-1

EIC of MS1 | MS1 spectra | MS2 - 1227.6>486.3 (y5+)

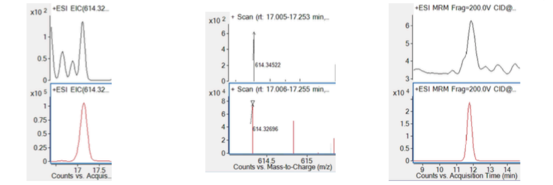

q. CAP-SIGLEC1

EIC of MS1 | MS1 spectra | MS2 - 843.9>799.9 (b16++)

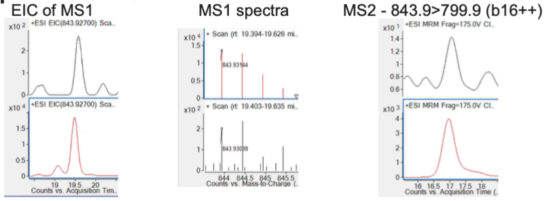

r. CAP-CPAMD8-2

EIC of MS1 | MS1 spectra | MS2 - 666.3>524.3 (a5+)

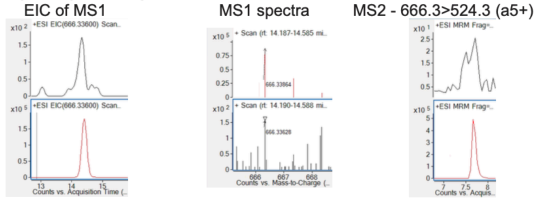

s. CAP-CBLN2

EIC of MS1 | MS1 spectra | MS2 - 788.4>717.4 (a14++)

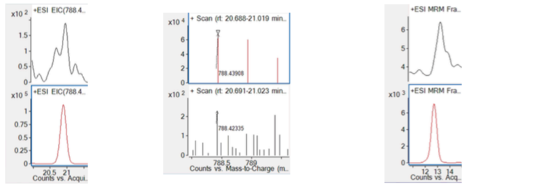

t. CAP-CPAMD8-1

EIC of MS1 | MS1 spectra | MS2 - 638.7>84.0 (a1+)

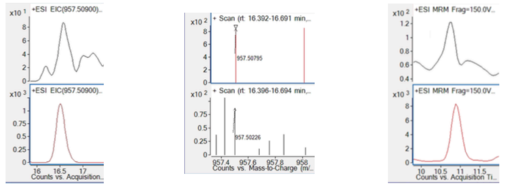

U. CAP-P.2MCH

EIC of MS1 | MS1 spectra | MS2 - 986.5>939.5 (b8+)

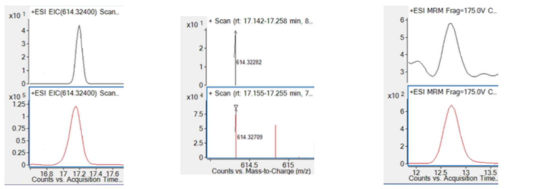

V. CAP-LEFTY2

EIC of MS1 | MS1 spectra | MS2 - 912.5>903.6 (b15+)

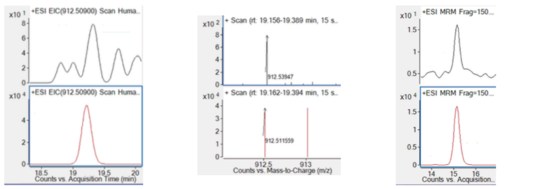

W. CAP-OSM

EIC of MS1 | MS1 spectra | MS2 - 597.7>146.1 (y1+)

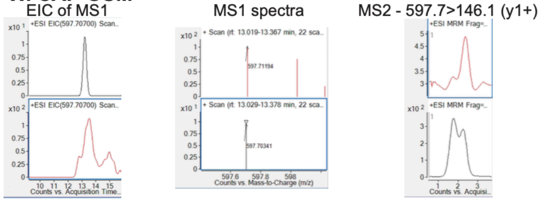

X. CAP-COL27A1

EIC of MS1 | MS1 spectra | MS2 - 493.3>212.1 (y2+)

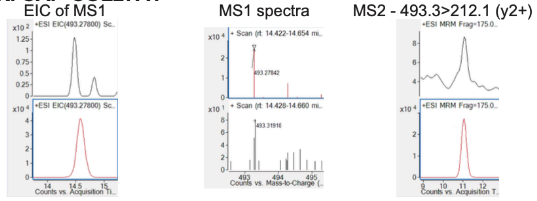

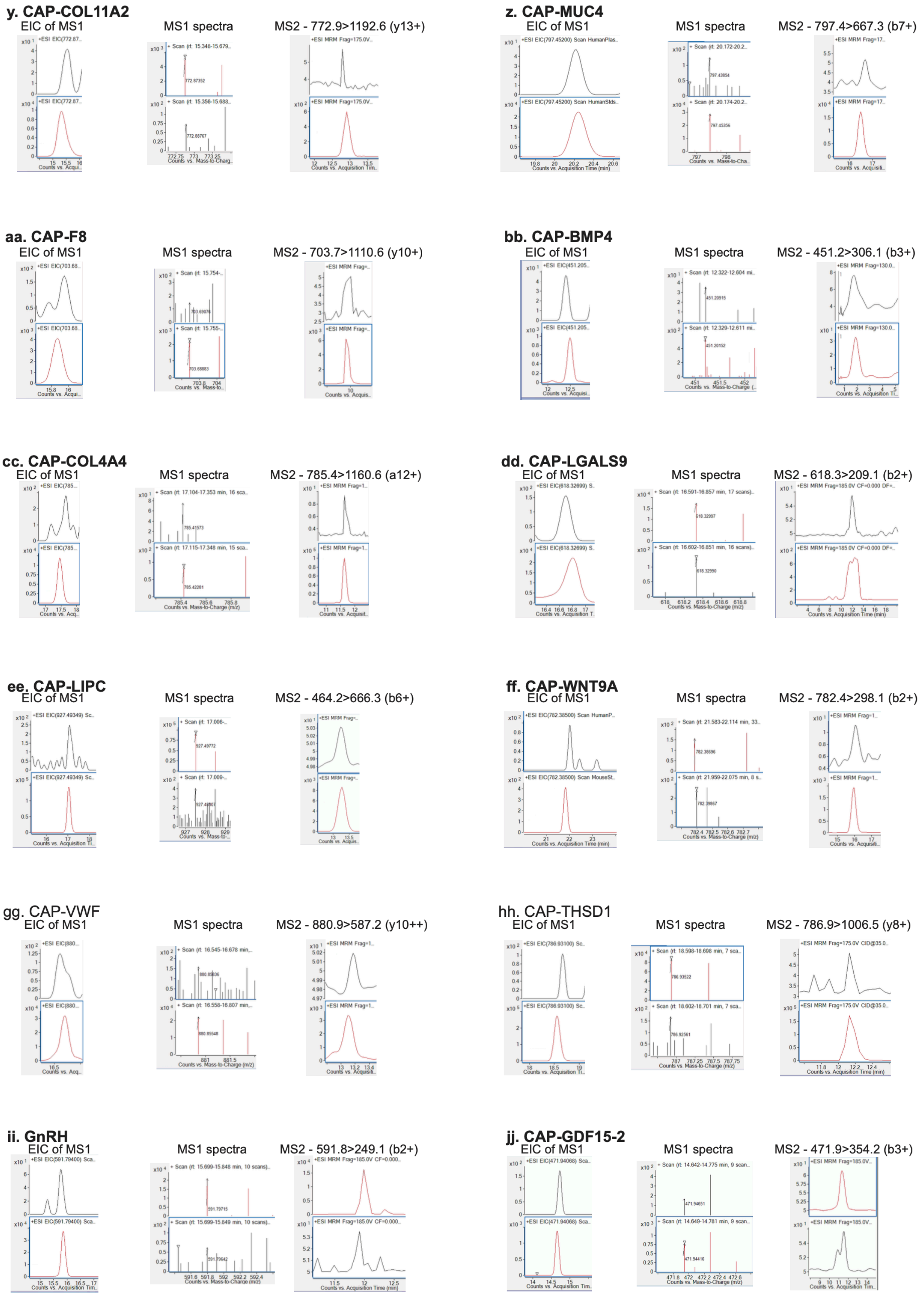
y. CAP-COL11A2
EIC of MS1
MS1 spectra
MS2 - 772.9>1192.6 (y13+)
z. CAP-MUC4
EIC of MS1
MS1 spectra
MS2 - 797.4>667.3 (b7+)
aa. CAP-F8
EIC of MS1
MS1 spectra
MS2 - 703.7>1110.6 (y10+)
bb. CAP-BMP4
EIC of MS1
MS1 spectra
MS2 - 451.2>306.1 (b3+)
cc. CAP-COL4A4
EIC of MS1
MS1 spectra
MS2 - 785.4>1160.6 (a12+)
dd. CAP-LGALS9
EIC of MS1
MS1 spectra
MS2 - 618.3>209.1 (b2+)
ee. CAP-LIPC
EIC of MS1
MS1 spectra
MS2 - 464.2>666.3 (b6+)
ff. CAP-WNT9A
EIC of MS1
MS1 spectra
MS2 - 782.4>298.1 (b2+)
gg. CAP-VWF
EIC of MS1
MS1 spectra
MS2 - 880.9>587.2 (y10++)
hh. CAP-THSD1
EIC of MS1
MS1 spectra
MS2 - 786.9>1006.5 (y8+)
ii. GnRH
EIC of MS1
MS1 spectra
MS2 - 591.8>249.1 (b2+)
jj. CAP-GDF15-2
EIC of MS1
MS1 spectra
MS2 - 471.9>354.2 (b3+)

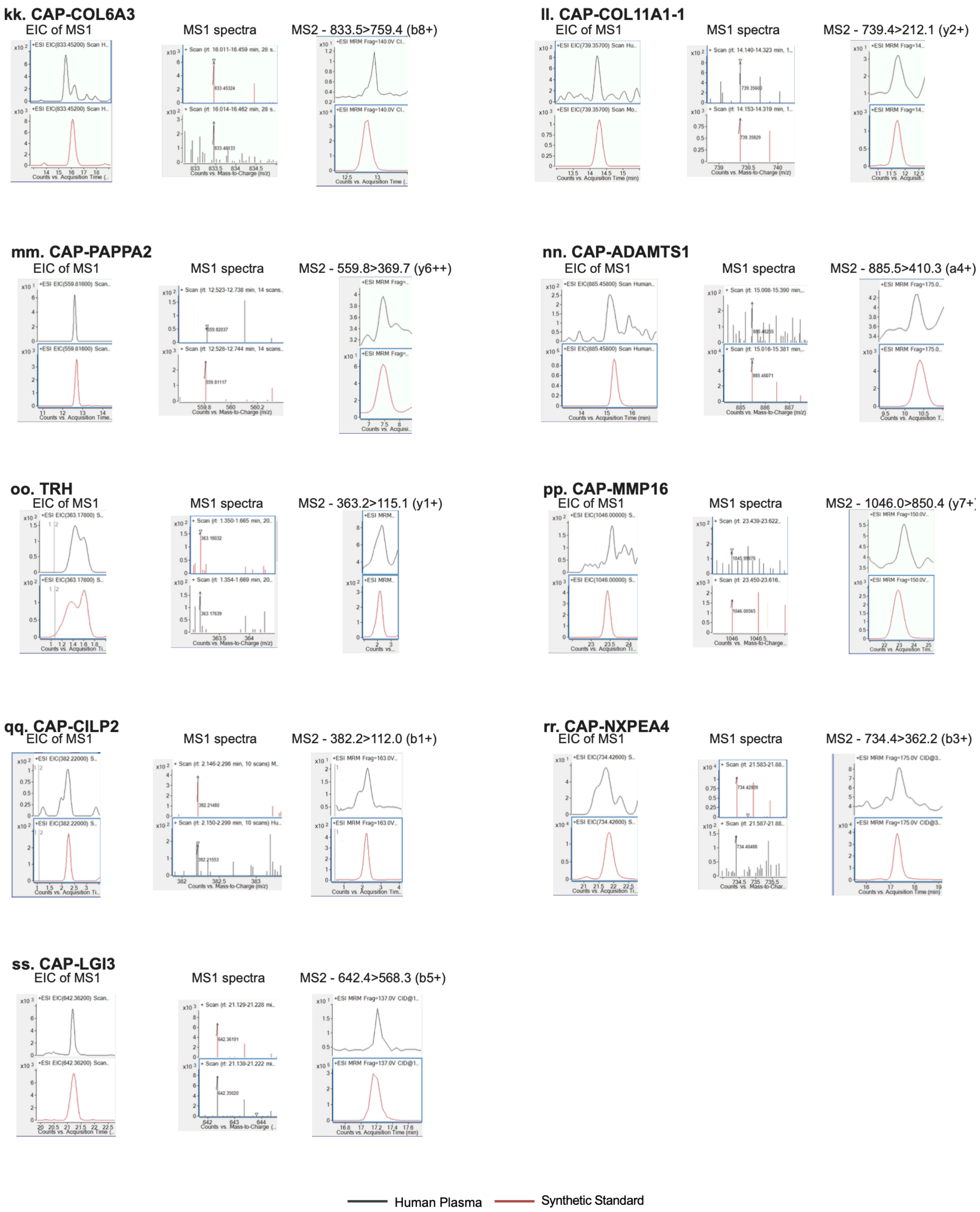

Supplementary Figure 6. All raw MS1 chromatograms, MS1 spectra, and MRM transition chromatograms for detected human capped peptides. a-ss The extracted parent ion chromatogram, the MS1 spectra, and MRM fragment ion chromatogram both in human plasma (black) and of the synthetic standards (red).

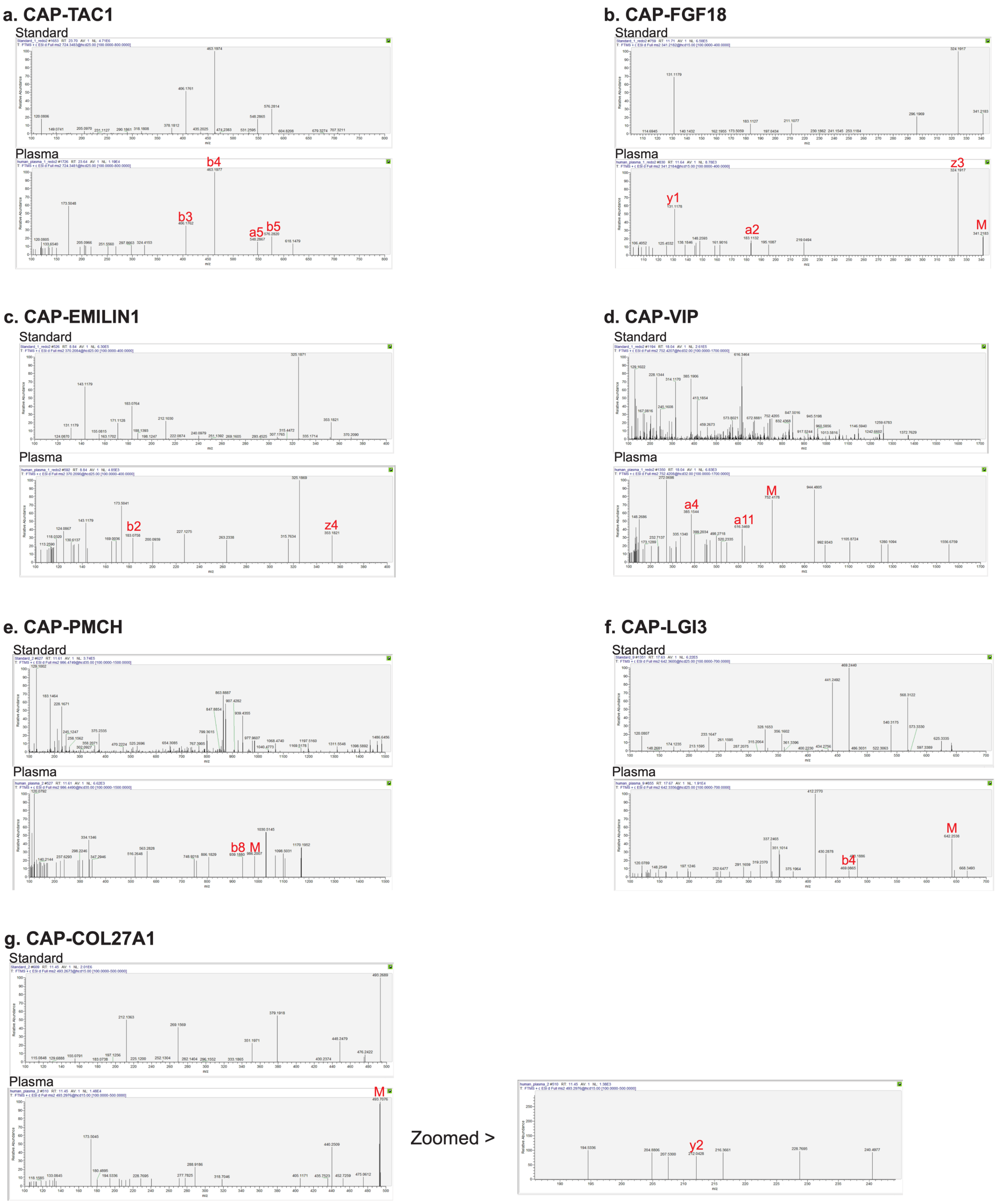
a. CAP-TAC1
Standard
Plasma
b4
b3
a5 b5
b. CAP-FGF18
Standard
Plasma
z3
y1
a2
M
c. CAP-EMILIN1
Standard
Plasma
b2
z4
d. CAP-VIP
Standard
Plasma
M
a4
a11
e. CAP-PMCH
Standard
Plasma
b8 M
f. CAP-LGI3
Standard
Plasma
M
b4
g. CAP-COL27A1
Standard
Plasma
M
Zoomed >
y2

h. CAP-COL11A2

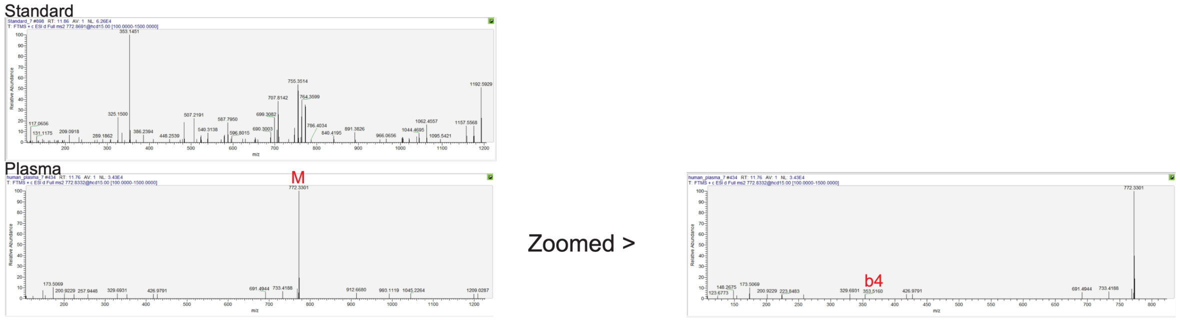

i. CAP-FURIN

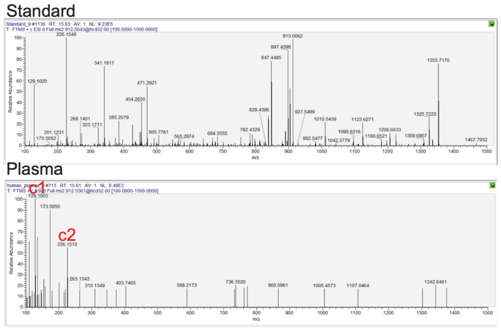

Supplementary Figure 7. Additional MS2 spectra for human capped peptides. a-i MS2 spectra of 9 human capped peptides both in human plasma and of the synthetic standards.

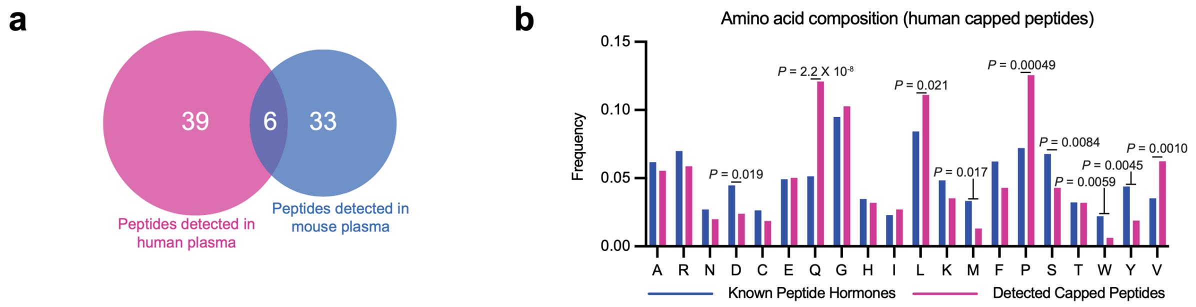

Supplementary Figure 8. Prediction, detection, and composition analysis of human capped peptides. a Venn diagram showing the amount of peptides detected in human and mouse plasma and overlap between the two. **b** Comparison of frequency of each amino acid between human capped peptides and known peptide hormones. For (**b**), data is presented as mean of frequency, and P-value versus control was calculated by by two-sided Student’s t-test. Source data are provided as a Source Data file.

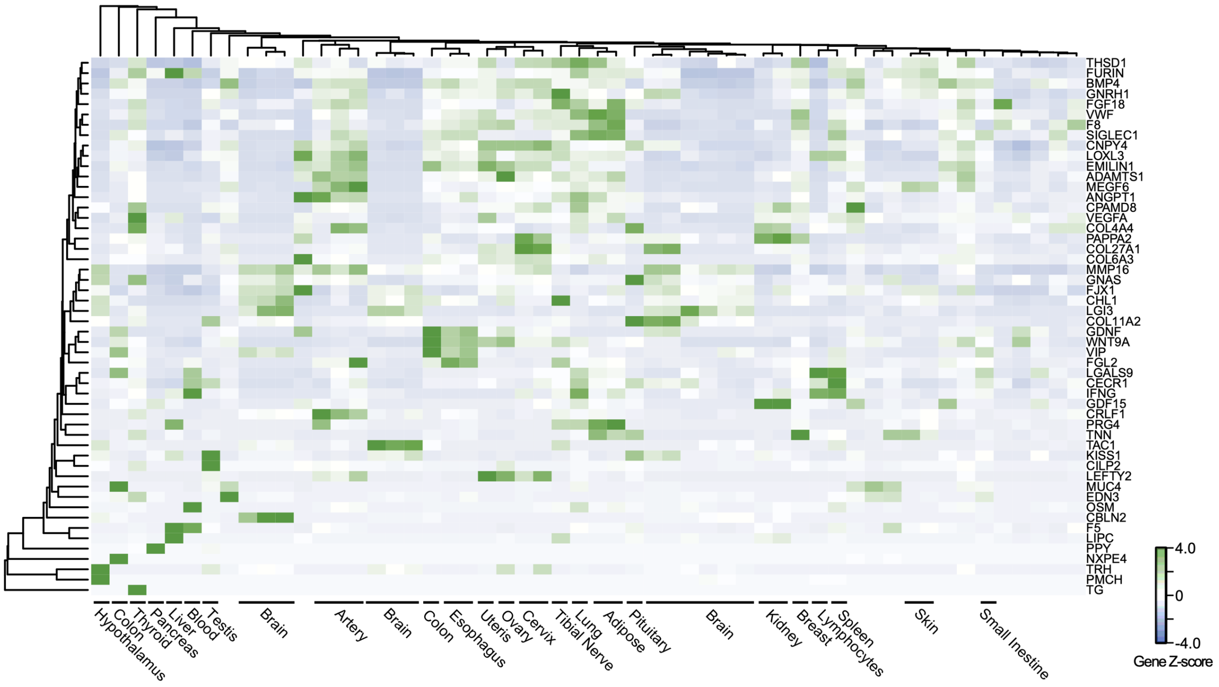

Supplementary Figure 9. Tissue distribution of mRNAs for home genes corresponding to human capped peptide. H-clustered heat map of mRNA expression for capped peptide preprop recursor home genes across human tissues and cell types, using GTEx as the reference databa se. Source data are provided as a Source Data file.
