# Supplementary material for: A class of secreted mammalian peptides with potential to expand cell-cell communication
Source: Nat Commun. 2023 Dec 8;14:8125. doi: 10.1038/s41467-023-43857-0 (PMC10709327; doi:10.1038/s41467-023-43857-0)
Supplement: Supplementary file 8 — Reporting Summary [file 41467_2023_43857_MOESM8_ESM.pdf]

Corresponding author(s): Jonathan Z Long
Last updated by author(s): Oct 30, 2023

Reporting Summary

Nature Portfolio wishes to improve the reproducibility of the work that we publish. This form provides structure for consistency and transparency in reporting. For further information on Nature Portfolio policies, see our Editorial Policies and the Editorial Policy Checklist.

Statistics

For all statistical analyses, confirm that the following items are present in the figure legend, table legend, main text, or Methods section.

n/a	Confirmed	
☐	☒	The exact sample size (*n*) for each experimental group/condition, given as a discrete number and unit of measurement
☐	☒	A statement on whether measurements were taken from distinct samples or whether the same sample was measured repeatedly
☐	☒	The statistical test(s) used AND whether they are one- or two-sided<br>*Only common tests should be described solely by name; describe more complex techniques in the Methods section.*
☒	☐	A description of all covariates tested
☒	☐	A description of any assumptions or corrections, such as tests of normality and adjustment for multiple comparisons
☐	☒	A full description of the statistical parameters including central tendency (e.g. means) or other basic estimates (e.g. regression coefficient) AND variation (e.g. standard deviation) or associated estimates of uncertainty (e.g. confidence intervals)
☐	☒	For null hypothesis testing, the test statistic (e.g. *F*, *t*, *r*) with confidence intervals, effect sizes, degrees of freedom and *P* value noted<br>*Give P values as exact values whenever suitable.*
☒	☐	For Bayesian analysis, information on the choice of priors and Markov chain Monte Carlo settings
☒	☐	For hierarchical and complex designs, identification of the appropriate level for tests and full reporting of outcomes
☒	☐	Estimates of effect sizes (e.g. Cohen's *d*, Pearson's *r*), indicating how they were calculated

Our web collection on statistics for biologists contains articles on many of the points above.

Software and code

Policy information about availability of computer code

Data collection	LC-QTOF and LC-QQQ data was collected with Agilent MassHunter Acquisition software. Columbus Instruments Oxymax/CLAMS software was used to collect data from metabolic chambers.
Data analysis	Heat maps and h-clustering was performed with the heatmap() function in the gplots R package. Other figures and statistical tests were performed in Prism GraphPad. LC-QTOF and LC-QQQ data were analyzed in Agilent MassHunter Qualitative software. Multiple sequence alignment was done with EMBL-EBI softwae and phylotree was made with ITOL. Code for capped peptide prediction was deposited to GitHub DOI: 10.5281/zenodo.8475 [https://github.com/amandawigg/Capped-Peptides/tree/capped-peptide#capped-peptides].

For manuscripts utilizing custom algorithms or software that are central to the research but not yet described in published literature, software must be made available to editors and reviewers. We strongly encourage code deposition in a community repository (e.g. GitHub). See the Nature Portfolio guidelines for submitting code & software for further information.

Data

Policy information about availability of data

All manuscripts must include a data availability statement. This statement should provide the following information, where applicable:

- Accession codes, unique identifiers, or web links for publicly available datasets
- A description of any restrictions on data availability
- For clinical datasets or third party data, please ensure that the statement adheres to our policy

Secretome sequences were collected from Uniprot. Expression data were collected from BioGPS and GTEx. All raw mass spectrometry data in Agilent .d file format

were deposited to Mendeley Data (https://data.mendeley.com/v1/datasets/rcm9k9d2by/draft?preview=1).

Research involving human participants, their data, or biological material

Policy information about studies with human participants or human data. See also policy information about sex, gender (identity/presentation), and sexual orientation and race, ethnicity and racism.

Reporting on sex and gender	N/A
Reporting on race, ethnicity, or other socially relevant groupings	N/A
Population characteristics	N/A
Recruitment	N/A
Ethics oversight	N/A

Note that full information on the approval of the study protocol must also be provided in the manuscript.

Field-specific reporting

Please select the one below that is the best fit for your research. If you are not sure, read the appropriate sections before making your selection.

[x] Life sciences [ ] Behavioural & social sciences [ ] Ecological, evolutionary & environmental sciences

For a reference copy of the document with all sections, see nature.com/documents/nr-reporting-summary-flat.pdf

Life sciences study design

All studies must disclose on these points even when the disclosure is negative.

Sample size	Sample size is 2 or more for all experimental assays, >3 in most cases. The selection of sample sizes was guided by prior literature in the field of metabolism and proteomics that had conducted similar experiments and by the number of replicates sufficient to power detection of an effect. For in vitro experiments, sample sizes ranged from 2 to 5 biological replicates, while for animal experiments, sample sizes of greater than 5 were used. The exact numbers were pre-determined based on the experimental design, the availability of animals, and the animal housing conditions.
Data exclusions	The only data excluded was that in which mice shredded their food out of their food cages. Thus, the food weight could not be accurately measured.
Replication	All experiments were independently replicated at least 3 times. Food intake experiments were replicated in at least 2 independent experiments and results were combined into the figures shown.
Randomization	Mice were randomly assigned to treatment groups, separating cages when possible to eliminate cage-specific effects.
Blinding	In all experiments, blinding of investigators was not implemented due to the quantitative nature of the measurements. All samples and experiments were performed in the way regardless of group or treatment.

Reporting for specific materials, systems and methods

We require information from authors about some types of materials, experimental systems and methods used in many studies. Here, indicate whether each material, system or method listed is relevant to your study. If you are not sure if a list item applies to your research, read the appropriate section before selecting a response.

Materials & experimental systems

n/a	Involved in the study	
[x]	[ ]	Antibodies
[ ]	[x]	Eukaryotic cell lines
[x]	[ ]	Palaeontology and archaeology
[ ]	[x]	Animals and other organisms
[x]	[ ]	Clinical data
[x]	[ ]	Dual use research of concern
[x]	[ ]	Plants

Methods

n/a	Involved in the study	
[x]	[ ]	ChIP-seq
[x]	[ ]	Flow cytometry
[x]	[ ]	MRI-based neuroimaging

Eukaryotic cell lines

Policy information about cell lines and Sex and Gender in Research

Cell line source(s)	PathHunter beta-arrestin CHO-K1 , from Eurofins DiscoverX
Authentication	PathHunter GPCR cell lines were authenticated by response to addition of exogenous ligand
Mycoplasma contamination	All cell lines tested negative for mycoplasma by Eurofins DiscoverX
Commonly misidentified lines (See ICLAC register)	None

Animals and other research organisms

Policy information about studies involving animals; ARRIVE guidelines recommended for reporting animal research, and Sex and Gender in Research

Laboratory animals	Jackson laboratories, C57BL/6J, mice 2-5 months of age, or 24 months for aged mice, stock #000664 and #380050 for diet-induced obese mice
Wild animals	No wild animals used
Reporting on sex	We report the sex of the mice for each experiment in the figure legends.
Field-collected samples	No field-collected samples
Ethics oversight	Animal experiments were performed according to a procedure approved by the Stanford University Administrative Panel on Laboratory Animal Care.

Note that full information on the approval of the study protocol must also be provided in the manuscript.
